# FDG PET biomarkers for prediction of survival in metastatic melanoma prior to anti-PD1 immunotherapy

**DOI:** 10.1038/s41598-021-98310-3

**Published:** 2021-09-22

**Authors:** A. Flaus, V. Habouzit, N. De Leiris, J. P. Vuillez, M. T. Leccia, J. L. Perrot, N. Prevot, F. Cachin

**Affiliations:** 1 Nuclear Medecine Department, Saint-Etienne University Hospital, University of Saint-Etienne, Saint-Etienne, France; 2 Nuclear Medicine Department, East Group Hospital, Hospices Civils de Lyon, Lyon, France; 3 Nuclear Medecine Department, CHU Grenoble Alpes, University Grenoble Alpes, Grenoble, France; 4 Laboratoire Radiopharmaceutiques Biocliniques, University Grenoble Alpes, INSERM, CHU Grenoble Alpes, 38000 Grenoble, France; 5 Dermatology Department, CHU Grenoble Alpes, University Grenoble Alpes, Grenoble, France; 6 Dermatology Department, Saint-Etienne University Hospital, University of Saint-Etienne, Saint-Etienne, France; 7 Nuclear Medicine Department, Jean Perrin Cancer Center of Clermont-Ferrand, Clermont-Ferrand, France; 8 Service de Medecine Nucléaire, Hôpital Nord, CHU de Saint-Etienne, 42 055 Saint-Etienne, Cedex 2, France

**Keywords:** Prognostic markers, Cancer, Skin diseases, Molecular medicine

## Abstract

Our aim was to analyse whether biomarkers extracted from baseline ^18^F-FDG PET before anti-PD1 treatment contribute to prognostic survival information for early risk stratification in metastatic melanoma. Fifty-six patients, without prior systemic treatment, BRAF wild type, explored using ^18^F-FDG PET were included retrospectively. Our primary endpoint was overall survival (OS). Total metabolic tumoral volume (MTV) and forty-one IBSI compliant parameters were extracted from PET. Parameters associated with outcome were evaluated by a cox regression model and when significant helped build a prognostic score. Median follow-up was 22.1 months and 21 patients died. Total MTV and long zone emphasis (LZE) correlated with shorter OS and served to define three risk categories for the prognostic score. For low, intermediate and high risk groups, survival rates were respectively 91.1% (IC 95 80–1), 56.1% (IC 95 37.1–85) and 19% (IC 95 0.06–60.2) and hazard ratios were respectively 0.11 (IC 95 0.025–0.46), *P* = 0.0028, 1.2 (IC 95 0.48–2.8), *P* = 0.74 and 5.9 (IC 95 2.5–14), *P* < 0.0001. To conclude, a prognostic score based on total MTV and LZE separated metastatic melanoma patients in 3 categories with dramatically different outcomes. Innovative therapies should be tested in the group with the lowest prognosis score for future clinical trials.

## Introduction

Immunotherapy using immune checkpoint inhibitors (ICI) targeting programmed cell death 1 receptor (PD-1) is now a well-established treatment for patients with metastatic melanoma^1^. Indeed, clinical benefits and durability of treatment response outperform standard chemotherapy^2^.

However, only subsets of patients will benefit from it^1^: thus, we need to develop tools predicting patient survival after immunotherapy to prevent toxicities and hasten the introduction of more appropriate treatments when necessary. To answer this question different biomarkers have been analysed such as levels of programmed cell death ligand 1 (PDL1) expression^3^, presence of tumour infiltrating lymphocytes^4^, genetic mutations^5^ and inflammatory cytokines^6^. Currently none are clinically identified as helpful for patient selection^7^.

Adopting the quantitative imaging biomarkers approach is an interesting alternative^8^. Indeed, various quantitative features can be extracted from images such as pixel intensity, shape and texture^9^.

^18^F-labeled fluorodeoxyglucose positron emission tomography -computed tomography (^18^F FDG PET-CT) provides valuable functional information based on the increased glucose uptake and glycolysis of metastatic melanoma cells^10^. ^18^F FDG PET-CT is commonly used for the initial staging of metastatic melanomas.

For metastatic melanoma, previous studies in heterogenous cohorts of patients—treated either with anti-cytotoxic T lymphocytes associated protein (anti-CTLA 4)^11^ or anti-PD1^12^-have shown that total metabolic tumour volume (total MTV) from baseline ^18^F FDG PET-CT correlate with overall survival (OS). Similar results were found for non-small cell lung cancer patients treated by immunotherapy^13^. Moreover, textural feature from baseline ^18^F FDG PET-CT, such as tumour heterogeneity, were also linked with OS^14^. However, in this study total MTV was not computed, and authors included patients with various stages and different immunotherapies (anti-PD1 and anti-CTLA 4).

The aim of our work is to analyse whether biomarkers—extracted from baseline ^18^F FDG PET-CT performed prior to anti-PD1 treatment—can improve information on survival and prognosis in a highly selected cohort of patients with metastatic melanoma.

## Materials and methods

### Source of data

The hospital information system of two university hospitals (University hospital of Saint-Etienne and Grenoble) was questioned to identify metastatic melanoma patients treated with first-line anti-PD1 treatment and imaged with a ^18^F-FDG PET-CT scan before therapy between January 2016 and January 2019. Electronic clinical and radiological databases were used to obtain patient demographic details, clinical history, anatomopathological data, treatment data, clinical outcome and follow-up duration as well as ^18^F-FDG PET data.

The protocol was approved by the institutional medical ethics committee (IRB: IORG0007394) of Saint-Etienne (IRBN 842,020/CHUSTE) and all methods were carried out in accordance with relevant guidelines and regulations. Informed consent was obtained from all the patients to participate in the study according to national regulations.

### Patients

Out of sixty-four screened patients, fifty-six were included. Inclusion criteria were as follows: (1) biopsy-proven metastatic melanoma; (2) anti-PD1 antibodies were the first line of treatment, with no previous systematic therapy; (3) patients were B-RAF wild type; (4) pre-treatment ^18^F-FDG PET data were available. Eight patients were excluded: patients with no measurable disease or no significant FDG avid tumour (n = 6). It included patients presenting only brain metastases due to the impossibility of clear delineation of metabolic tumoral volume, and patients with lesion sizes inferior to 64 voxels (n = 2), the radiomic analysis becoming irrelevant below this threshold^15,16^.

Metastatic status was classified as defined by the eighth edition American Joint Committee on Cancer (AJCC) melanoma staging system^17^:

- M1a: distant metastasis to skin, soft tissue including muscles, and/or nonregional lymph
- M1b: distant metastasis to lung with or without M1a sites of disease
- M1c: distant metastasis to non-central nervous system (CNS) visceral sites with or without M1a or M1b sites of disease
- M1d: distant metastasis to CNS with or without M1a, M1b, or M1c sites of disease.

### Outcome: overall survival

OS was defined as the time (in months) between the ^18^F-FDG PET scan and the date of death of any cause. The length of the follow up was calculated starting from the date of the initial ^18^F-FDG PET up to the date of the last clinical consultation. Living patients were censored at the time of the last clinical follow-up.

### ^18^F-FDG PET-CT protocol

Scans were acquired with different PET-CT systems: Biograph mCT Flow 20 (Siemens Healthcare) and Biograph 6 HI-REZ (Siemens Healthcare) were used at the first University Hospital. Biograph Horizon 16 (Siemens Healthcare) and Discovery 690 (General Electrics Healthcare,) were employed at the second University Hospital.

Patients fasted for at least 6 h and blood glucose was < 180 mg/dl. Patients were injected according to current guidelines with an activity of 2.5–4 MBq/kg of ^18^F-FDG (median activity 269 MBq, range 146–468). Sixty minutes after injection whole-body PET and unenhanced CT images were acquired. Images were reconstructed using iterative algorithms and using CT for attenuation correction. As shown in Table 1, image reconstruction parameters were different but consistent for each PET scanner. Using the following formula, each voxel in PET images was converted to standard uptake value (SUV) : SUV = voxel concentration activity *patient body weight / decay corrected injected activity^18^.

**Table 1:** Image reconstruction parameters for all four different PET systems.

Scanner	Reconstruction	Iteration and subsets	Gaussian post-filtering	Matrix	Voxel size (x,y,z mm)
Siemens, Biograph Horizon 16	OSEM	3 iterations and 10 subsets	FWHM 4 mm	360	2.06 × 2.06 × 2.03
GE, Discovery 690	OSEM	2 iterations and 24 subsets	FWHM 4 mm	256	2.73 × 2.73 × 3.27
Siemens, Biograph 20 mCT	OSEM	2 iterations and 21 subsets	FWHM 5 mm	256	1.59 × 1.59 × 3
Siemens, Biograph 6 HI-REZ	OSEM	4 iterations and 8 subsets	FWHM 5 mm	256	4.06 × 4.06 × 4

*OSEM* ordered subsets expectation maximization, *FWHM* full width at half maximum.

### Measuring imaging biomarkers

We performed segmentation and the quantitative features’ extraction process using the free software LIFEx (v4.0, Local Image Feature Extraction) which complies with the image biomarker standardization initiative (IBSI)^16,19^. One experienced nuclear medicine physician analysed and segmented ^18^F-FDG PET scans without knowledge of the patients’ clinical outcome. Based on a 40% SUVmax threshold, a semi-automatic method was employed for segmentation^20^.

#### Total metabolic tumour volume

Each hypermetabolic lesion was segmented to create a volume of interest (VOI) corresponding to the MTV. SUVmean was measured as the average uptake in the tumour VOI. A total MTV per patient was defined as the sum of the MTV of each hypermetabolic metastatic lesion^11,12^.

#### Conventional and Textural PET parameters

For each patient, quantitative features were extracted from the VOI of the lesion with the highest ^18^F-FDG uptake. Before textural feature calculation, voxel intensities of the VOI were resampled using 64 discrete values ranging from 0 to 32 SUV units^21^. 41 features in total were extracted: five conventional PET parameters—SUVmax, SUVmean, SUVmin, SUVpeak and Total lesion glycolysis (TLG)—five descriptors of the image intensity histogram, and 31 s order textural features—six from the Grey Level Co-Occurrence matrix (GLCM), eleven from the Grey-level run length matrix (GLRLM), three from the Neighbourhood grey-level different matrix (NGLDM) and eleven from the Grey-level zone length matrix (GLZLM). A detailed description of each parameter is available in the technical appendix of the LIFEx software^16^.

### Feature pre-processing

As the study was retrospective no pre acquisition harmonisation was performed. However, in order to pool together conventional and textural features from the four different PET/CT scanners, ComBat post-reconstruction harmonization method was used^22,23^. After ComBat harmonization, a feature selection was used to shrink the model, reducing overfitting and co-variate correlation. Features’ bilateral correlation were evaluated with Spearman’s rank correlation coefficient^24^ and those with correlation coefficient higher than 0.8 were removed^25^.

### Statistical analysis

For statistical analyses and plotting the open-source *R* software package was utilized^26^ (version 3.0.1, http://www.Rproject.org). Continuous variables are presented as median [range] and were compared using Wilcoxon rank sum test. Discrete variables are presented as number and percentage and were compared using chi-test or fisher-test if the amount of data were below 5.

In order to select PET parameters for further analysis, we compared mean values of pre-treatment PET parameters according to OS status at the time of analysis using two sided Wilcoxon—Mann Whitney tests because these parameters did not follow a Gaussian distribution (*P* < 0.0035 was considered as statistically significant after Bonferroni correction). Only those that were statistically significant were selected for further analysis and optimal cut-off for each significant parameters was based on the receiver operating characteristic (ROC) curves (pROC^27^ package, version 1.17.0.1, http://expasy.org/tools/pROC/).

Selected PET biomarkers, age, gender, and metastatic status were included in univariate and multivariate Cox regression models. Because of the small number of events, the multivariable analysis was restricted to variables that displayed a univariate analysis’ Hazard Ratio (HR) with a p value below 0.05. Kaplan Meier curves for OS were built for variables found significant during the multivariate analysis and compared with log-rank tests (*P* < 0.05 was considered statistically significant). Survival rates were calculated for each group at the end of the follow-up period (Survival package, version 3.2–7, https://CRAN.R-project.org/package=survival).

With the aim of stratifying the population into different risk categories, we constructed a prognostic composite metabolic score based on the parameters we found significant in the Cox models. Kaplan Meier analyses were computed for these different risk groups and compared with log-rank test (*P* < 0.05 was considered statistically significant).

## Results

### Patient characteristics

Pre-treatment patient characteristics are summarized in Table 2. Fifty-six patients with wild-type BRAF metastatic melanoma were included with a median age of 68 years old (range: 40–84). They all received first-line anti-PD1 therapies (Nivolumab or Pembrolizumab) as their first systemic treatment. At the time of analysis, the median follow-up was 22.1 months (range: 2.1–49.2) and 21 patients (38%) had died. There were no statistically significant differences between groups of patients alive and dead at time of analysis about sex (*P* = 0.3), age (*P* = 0.99), university hospital (*P* = 0.94), metastatic status before the start of anti-PD1 treatment (*P* = 0.91), anatomopathological characteristic of initial melanoma lesion such as Breslow (*P* = 0.56), presence of ulceration (*P* = 0.81), high rate of mitosis (> 1/mm^2^) (*P* = 0.97), localisation (*P* = 0.88) and the initial cancer staging (*P* = 0.57) (Supplementary Table 2).

**Table 2:** Clinical characteristics of the patients (n = 56).

Characteristics	Median[range], n(%)
**Clinical characteristics**	
Demographic parameters	
Age (years)	68 [40–84]
Female	27 (48%)
Male	29 (52%)
**PET Centre**	
Saint-Etienne PET Centre	33 (59%)
Siemens, Biograph 6 HI-REZ	14 (25%)
Siemens, Biograph 20 mCT	19 (34%)
Grenoble PET Centre	23 (41%)
GE, Discovery 690	12 (21%)
Siemens, Biograph Horizon 16	11 (20%)
**Metastatic status (8th AJCC classification)**	
M1a	13 (23%)
M1b	5 (9%)
M1c	31 (55%)
M1d	7 (13%)
**Treatment**	
Median duration between PET and start of treatment (months)	1.1 [0.03–9.07]
**Follow-up**	
Duration (months)	22.1 [2.1–49.2]
**Survival**	
Death	21 (38%)

*SUV* standardized uptake value, *PET* positron emission tomography, *M* Metastatic status (8th American Joint Committee on Cancer classification):

M1a: distant metastasis to skin, soft tissue including muscles, and/or nonregional lymph.

M1b: distant metastasis to lung with or without M1a sites of disease.

M1c: distant metastasis to non-central nervous system (CNS) visceral sites with or without M1a or M1b sites of disease.

M1d: distant metastasis to CNS with or without M1a, M1b, or M1c sites of disease.

### PET biomarkers correlated with survival

**Figure 1:**
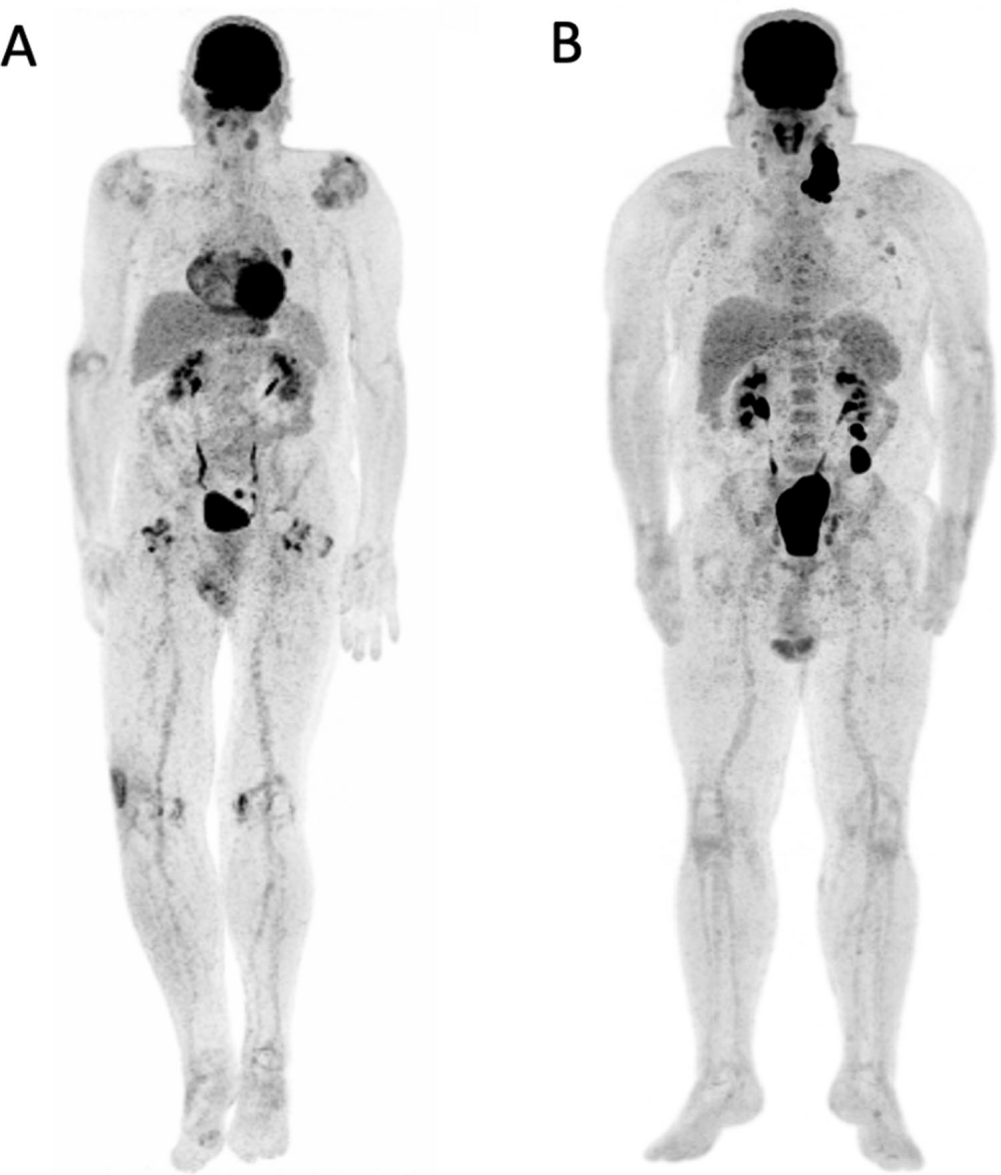
The maximum intensity projection PET images from two subjects. (**A**) Alive at the time of analysis, showed an inguinal right lymph node and a left pulmonary nodule (total MTV 3 cm^3^) with low LZE (− 4194) and (**B**) dead at the time of analysis, showed cervical, axillary, celiac, mesenteric, iliac lymph nodes (total MTV 50,5 cm^3^) with high LZE (58,336). Total MTV: total metabolic tumoral volume, LZE: long zone emphasis.

We correlated ^18^F-FDG PET biomarkers with OS status in patients treated with anti-PD1. Only mean total MTV and mean Long Zone Emphasis (LZE) differed significantly between patients that were alive or dead at the time of the analysis (Supplementary Table 1). Mean total MTV burden was significantly lower in the group of patients alive at the time of analysis (mean 18.22 cm^3^, IC95 7.08–29.36 vs. 49.69 cm^3^, IC95 16.88–82.49; *P* = 0.001). To identify patients with the worse prognosis, the optimal mean total MTV cut-off value was 5.6 cm^3^, with a sensitivity of 0.9 and a specificity of 0.63 (ROC curve AUC = 0.76, CI95 0.64–0.89; Supplementary Fig. 1a). Figure 1 represents maximum intensity projection PET images from two subjects. (A) alive at the time of analysis with low total MTV (3 cm^3^) and low LZE (− 4194). (B) dead at the time of analysis with high total MTV (50,5) and high LZE (58,336). Moreover, mean LZE, extracted from the GLZLM matrix, was significantly lower in the group of patients alive at the time of analysis (− 1040.60, CI95 − 5615.21–3535 vs. 10,507.6, CI95 2309.75–18,705.44, *P* = 0.003). The optimal mean LZE cut-off value to identify worse prognostic patients was–437, with a sensitivity of 0.62 and a specificity of 0.89 (ROC curve AUC = 0.73, CI95 0.57–0.89; Supplementary Fig. 1b).

In univariate analysis, only high total MTV (*P* = 0.002) and high LZE (*P* < 0.001) were significantly correlated with poor OS. No clinical parameters were significantly associated with the outcome (Table 3). Looking at the multivariate analysis, high total MTV and high LZE remained statistically significant independent prognostic factors for OS (HR 4.1 CI95 1.1–15.3, *P* = 0.034 and HR 3.7 CI95 1.5–9.5, *P* = 0.006, respectively) (Table 3). Using log rank test, Kaplan Meier curves comparing overall survival in patients with high versus low total MTV and high versus low LZE were significantly different, with respective p values of 0.00038 and < 0.0001 (Supplementary Fig. 2). Survival rates were 87.6% (CI95 75.4–1) for low MTV versus 40.3% for high MTV (CI 95 25.9–62.8). Survival rates were 77% (CI 95 64.8–91.4) for low LZE versus 55.2% for high LZE (CI 95 7.1–61.4).

**Table 3:** Univariate and multivariate cox regression analyses of overall survival in patients with metastatic melanoma.

Variable	Univariate analysis			Multivariate analysis		
	HR	95% CI	*p*-Value	HR	95% CI	*P*-value
Age	1	1–1	0.96	–	–	–
Sex	0.65	0.3–1.6	0.33	–	–	–
M status				–	–	–
M1a vs. M1b	0.4	0.05–3.4	0.8			
M1a vs. M1c	0.85	0.3–2.4	0.9			
M1a vs. M1d	0.97	0.2–4	0.9			
LZE (GLZLM)	6.4	2.6–16	< 0.001	3.7	1.5–9.5	0.006
Total MTV	6.3	1,9–21	0.003	4.1	1.1–15.3	0.034

The distribution of each ^18^F-FDG PET biomarker is a continuous variable that is transformed into a discrete categorization of 2 categories (high vs. low) using the values derived from the Youden’s index: LZE (≥ − 437 vs < − 437), total MTV (≥ 5.6 vs < 5.6 cm^3^).

*HR* hazard ratio, *CI* confidence interval, LZE (GLZLM) Long zone emphasis from the grey-level zone length matrix long-zone, *MTV* metabolic tumoral volume, *NGLDM* Neighbourhood grey-level different matrix, *TLG* total lesion glycolysis, *M* Metastatic status (8th American Joint Committee on Cancer classification):

M1a: distant metastasis to skin, soft tissue including muscles, and/or nonregional lymph.

M1b: distant metastasis to lung with or without M1a sites of disease.

M1c: distant metastasis to non-central nervous system (CNS) visceral sites with or without M1a or M1b sites of disease.

M1d: distant metastasis to CNS with or without M1a, M1b, or M1c sites of disease.

### Prognostic composite metabolic score

Since total MTV and LZE are both independent prognostic factors for OS in our multivariate analysis, we developed a prognostic score by combining them. The population was therefore stratified in three risk categories: low risk if total MTV ≤ 5.6 cm^3^ and LZE ≤ − 437 (n = 23; 41%), intermediate risk if total MTV > 5.6 cm^3^ or LZE > − 437 (n = 19, 34%) and high risk if total MTV > 5.6 cm^3^ and LZE > − 437 (n = 14; 25%).

Survival rates were respectively 91.1% (CI 95 80–1) for the low risk group, 56.1% for the intermediate risk group (CI 95 37.1–85) and 19% for the high risk group (CI 95 0.06–60.2). HR for OS were respectively 0.11 for the low risk group (CI95 0.025–0.46), *P* = 0.0028, 1.2 for the intermediate risk group (CI 95 0.48–2.8), *P* = 0.74 and 5.9 for the high risk group (CI 95 2.5–14), *P* < 0.0001.

**Figure 2:**
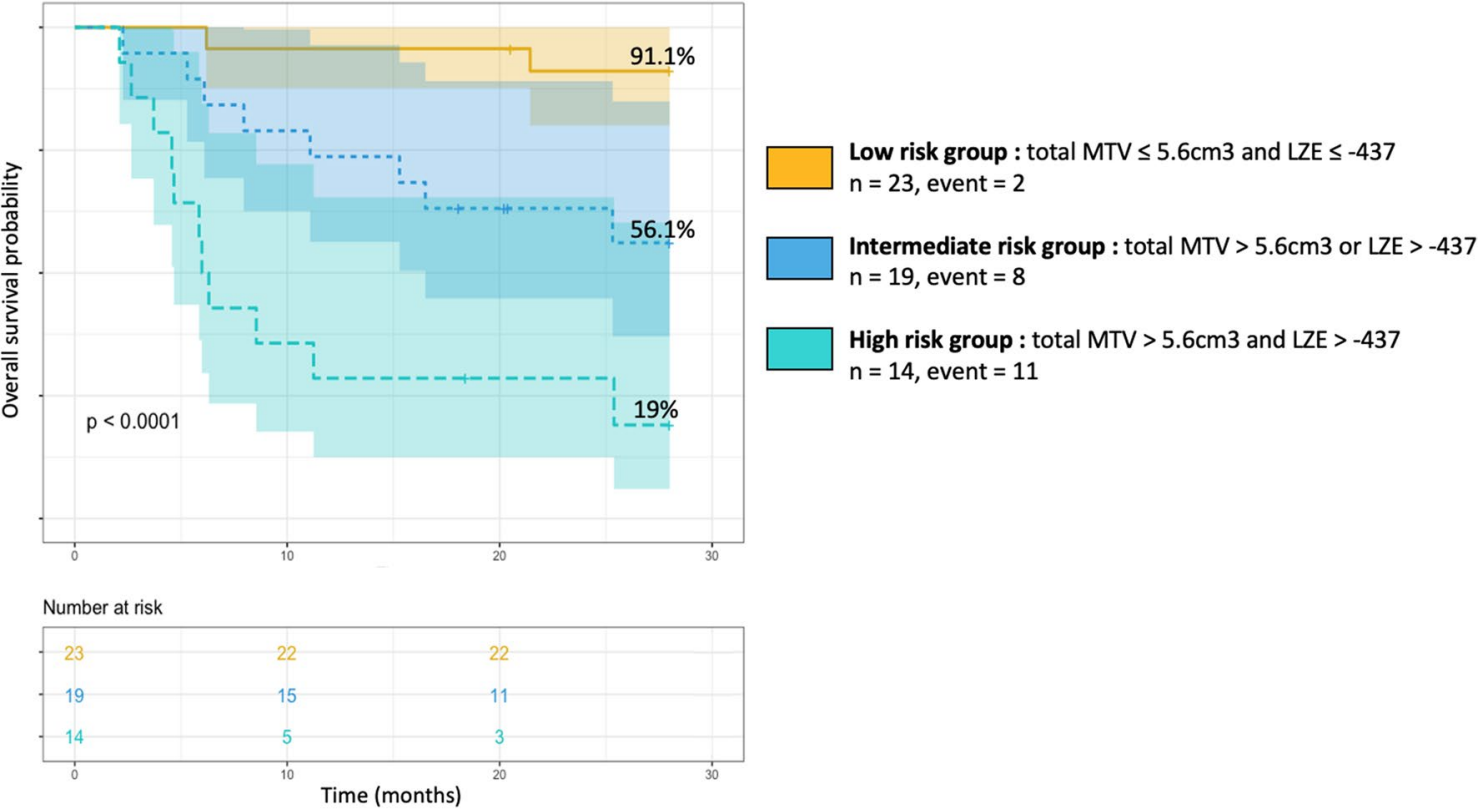
Kaplan Meier curves of overall survival (OS) based on a metabolic score combining total metabolic tumoral volume (total MTV) and long zone emphasis (LZE). The combined score comprising two binary risk variables was defined as follows: 0 risk variable = low risk group (total MTV ≤ 5.6 cm^3^ and LZE ≤ − 437), 1 risk variable = intermediate risk group (total MTV > 5.6 cm^3^ or LZE > − 437) and 2 risk variables = high risk group (total MTV > 5.6 cm^3^ and LZE > − 437).

Figure 2 represents Kaplan Meier curves of OS according to the three risk categories (*P* < 0.0001). Log rank tests differed significantly between Kaplan Meier curves for OS of low versus intermediate-risk groups (*P* = 0.008), low versus high-risk groups (*P* < 0.001), and intermediate versus high-risk groups (*P* = 0.013) (Supplementary Fig. 3).

## Discussion

Our results add to the accumulating evidence that beyond initial staging, ^18^F-FDG PET-CT scan provides simple to use and cost-effective biomarkers associated with OS. LZE and total MTV demonstrated their high prognostic value in metastatic melanoma patients treated with anti-PD1. As the former was highly specific, the latter highly sensitive, and they were not correlated, we used both parameters to build three risk categories, creating a prognostic composite metabolic score. For low, intermediate and high-risk groups, survival rates were respectively 91.1% (CI 95 80–1), 56.1% (CI 95 37.1–85) and 19% (CI 95 0.06–60.2). These imaging metrics provide a novel method for improving the stratification and selection of patients who would most benefit from additional immunotherapies beyond anti-PD1.

In our study, patients with a high metabolic tumour burden (total MTV > 5.6 cm^3^) exhibited a poorer prognostic, with a shorter median OS, strengthening the prognostic significance of pre-treatment total MTV before anti-PD1 therapies^28^. Previous studies found similar results in heterogeneous cohorts of melanoma patients, irrespectively of their BRAF status. In the first study, 55 patients underwent ^18^F-FDG PET before anti-PD1 as a first or further line treatment : high total MTV on baseline imaging was significantly correlated with a shorter survival (in multivariable analysis) with a cut-off value set at 25 cm^3^^12^. In a second retrospective cohort, 142 patients underwent ^18^F-FDG PET before anti-CTL4 as a first line or further line treatment : high total MTV was significantly correlated with OS (after multivariate analysis), with a cut-off value set at 26.85 cm^3^^11^. In our study, the total MTV cut-off was smaller because our population was limited to patients who received anti-PD1 as a first line therapy. To the best of our knowledge, our study is the first to analyse the prognostic value of total MTV in this specific sub-group of patients with metastatic melanoma, thus extending its validity as a prognostic biomarker.

Textural feature, LZE, brought additional prognostic information as it allowed a reclassification of patients into an intermediate risk group (total MTV > 5.6 cm^3^ or LZE > − 437) which encompassed 34% of the population (n = 19). Seventeen of these nineteen patients exhibited a high total MTV. No correlation was found between total MTV and LZE values, suggesting that LZE status dichotomize high total MTV patients in two sub-groups with a different prognosis.

LZE measures the distribution of long homogeneous grey level zones in the image irrespectively of their intensity: therefore, LZE reflects the lesion’s heterogeneity. In our study, patients with high value of tumoral LZE showed poorer survival. Despite this promising finding, it is difficult to interpret the underlying mechanism translated by this heterogeneity parameter, which is based on a mathematical equation. Further investigations are required to understand the biological mechanisms underlying these heterogeneity parameters.

A metabolic score combining total MTV and LZE enhanced the prognostic value of ^18^F-FDG PET imaging. Three different prognostic groups were identified. Low risk patients represented 41% of the population. Survival rate was 91.1% (CI 95 80–1) suggesting it is a biomarker for favourable outcome for anti-PD1 treatment. On the contrary, high-risk patients (25%) had a poorer prognosis since their survival rate was 19% (CI 95 0.06–60.2). So, our composite score appears as a biomarker, identifying patients who will not benefit from anti-PD1 therapy. This promising and innovative score provides the proof of concept for treatment personalization and should be validated in a larger, independent and prospective cohort.

Previous studies suggested that textural parameters from ^18^F-FDG PET-CT could predict outcome in various cancer types^29–32^ as it might represent tumour microenvironment, a known factor correlating with patients’ response to anti-PD1 and anti-PDL1^33–35^. Few studies in heterogeneous cohorts have investigated the potential for radiomics-extracted from ^18^F-FDG PET images-to predict outcomes in metastatic melanoma after immunotherapy, and their results are contradictory^12,14,36^. For one study, the predictive value of SUV heterogeneity did not show prognostic value in an heterogeneous cohort of 55 melanoma patients treated by anti-PD1^12^. However, in other studies textural parameters do carry prognostic information: in 34 patients with melanoma (stage from I to IV) treated with various immunotherapies (anti-PD1 and anti-CTLA4) as first or other line of treatment, gradient based tumour heterogeneity on pre-treatment ^18^F-FDG PET correlated to survival outcome, as well as SUVmax and TLG. However as it was not compliant with the IBSI, comparison was limited^14^. Moreover, another study found a positive association between second order features and treatment response in 17 advanced melanoma patients treated respectively with anti-CTLA4 or Vemurafenib (PLX4032) according to their BRAF V600E status^36^. In line with this study, we correlated LZE, a second order feature, with overall survival in patient with metastatic melanoma that were naïve of any previous systemic therapy prior to anti PD1. These data suggest that textural features, which are easily computed by free software, should be systematically assessed in prospective studies including ^18^F FDG PET-CT.

Our study has several limitations. Firstly, its design was retrospective and relatively few patients were included. Secondly, textural features exhibit varying sensitivity to the acquisition and reconstruction parameters and have previously limited multi PET scanner studies^37^. However in our work, the validated Combat harmonisation method was used^22,23^ to correct the effect of multi PET scanner as in other recent studies^30,38^. This method has proved its robustness even for a small number of patients per batch^39^. Thirdly the cut-offs that we used to create the 3 different risk groups are probably relying on PET acquisition system and reconstruction parameters. So it would be necessary to validate their robustness in a multicentric and prospective study. Lastly, as the study cohort was relatively small compared to the numbers of extracted features, Type 1 error rate may have increased. To prevent this, parameters highly inter-correlated were removed, thus decreasing redundant information, and we kept only independent parameters (n = 14 vs. n = 42).

To conclude, we built a highly prognostic composite metabolic score based on LZE and total MTV values from baseline ^18^F FDG PET and used it to predict overall survival prior anti-PD1 therapy in a highly selected cohort of metastatic melanoma patients with no previous systemic treatment. This model could be implemented in clinical trials and then in clinical practice to stratify patients before treatment, because it represents a valuable biomarker that identifies patients who will not benefit from anti-PD1 therapy and therefore should switch to other treatment modalities.

## Supplementary Information

Supplementary Information.

## Data Availability

The datasets used and/or analysed during the current study are available from the corresponding author upon reasonable request.
