# Supplementary material for: FDG PET biomarkers for prediction of survival in metastatic melanoma prior to anti-PD1 immunotherapy
Source: Sci Rep. 2021 Sep 22;11:18795. doi: 10.1038/s41598-021-98310-3 (PMC8458464; doi:10.1038/s41598-021-98310-3)
Supplement: Supplementary file 1 — Supplementary Information. [file 41598_2021_98310_MOESM1_ESM.pdf]

Supplementary data

Title: FDG PET biomarkers for prediction of survival in metastatic melanoma prior to anti-PD1 immunotherapy

Authors: A FLAUS MD[1, 2*], V HABOUZIT MD[1], N DE LEIRIS MD[3,4], J-PVUILLEZ MD PhD[3,4], M-T LECCIA MD PhD[5], J-L PERROT MD PhD[6], N PREVOT MD[1], F CACHIN MD PhD[7]

[1]Nuclear Medecine Department, Saint-Etienne University Hospital, University of Saint-Etienne, France

[2]Nuclear Medicine Department, East Group Hospital, Hospices Civils de Lyon, Lyon, France

[3]Nuclear Medecine Department, CHU Grenoble Alpes, Univ. Grenoble Alpes, France

[4]Univ. Grenoble Alpes, INSERM, CHU Grenoble Alpes, Laboratoire Radiopharmaceutiques Biocliniques, 38000 Grenoble, France

[5] Dermatology Department, CHU Grenoble Alpes, Univ. Grenoble Alpes, France

[6] Dermatology Department, Saint-Etienne University Hospital, University of Saint-Etienne, France

[7]Nuclear Medicine Department, Jean Perrin Cancer Center of Clermont-Ferrand, France

Uncorrelated PET parameters	p-value
CONVENTIONAL_SUVmin	0.084
CONVENTIONAL_TLG	0.019
HISTO_Skewness	0.52
HISTO_Entropy_log10	0.50
HISTO_Energy	0.98
GLCM_Correlation	0.20
GLRLM_SRE	0.60
GLRLM_LRE	0.33
GLRLM_LGRE	0.13
NGLDM_Coarseness	0.04
NGLDM_Contrast	0.50
NGLDM_Busyness	0.80
**GLZLM_LZE**	**0.003**
**Total MTV**	**0.001**

Supplementary Table 1. Uncorrelated conventional and textural PET parameters with respective p-value from Wilcoxon – Mann Whitney test between patients that were alive or dead at the time of the analysis.

SUV = Standardized uptake value, TLG = Total Lesion Glycolysis, HISTO = histogram, GLCM = Grey Level Co-Occurrence matrix, GLRLM = Grey-level run length matrix, NGLDM = Neighborhood grey-level different matrix, GLZLM = Grey-level zone length matrix, SRE = Short-Run Emphasis, LRE = Long-Run Emphasis, LGRE = Low Gray-Level Run Emphasis, LZE = Long Zone Emphasis.

Patients' characteristics	Dead at the time of analysis (n=21)	Alive at the time of analysis (n=35)	p-value
**Demographics**			
Sex (male)	9 (43%)	20 (57%)	0.3
Age	67.6[47-81]	68.5[25-89]	0.99
Saint-Etienne	14 (66.7%)	23 (65.7%)	0.94
**Metastatic status before immunotherapy treatment**			0.91
M1a	5 (23.8%)	8 (22.9%)	1
M1b	1 (4.7%)	4 (11.4%)	0.64
M1c	12 (57.1%)	19 (54.3%)	0.68
M1d	3 (14.3%)	4 (11.4%)	1
**Initial melanoma lesion**			
Anatomopathological characteristics			
Breslow			0.56
1	1 (4.7%)	5 (14.3%)	0.39
2	1 (4.7%)	4 (11.4%)	0.64
3	5 (23.8%)	7 (20%)	0.74
4	11 (52.4%)	12 (34.3%)	0.25
Presence of ulceration	12 (57.1%)	10 (28.6%)	0.81
Mitosis (> 1/mm2)	14 (66.7%)	21 (60%)	0.97
Localisation			0.88
Head and neck	6 (29%)	3 (8.6%)	1
Lower limb & hip	9 (43%)	6 (17.1%)	1
Upper limb and shoulder	8 (38%)	5 (14.3%)	1
Torso	1 (4.7%)	1 (2.9%)	1
Back	5 (23.8%)	2 (5.7%)	1
Acral	2 (9.5%)	2 (5.7%)	1
**Initial cancer staging**			0.57
1	8 (38%)	4 (11.4%)	1
2	11 (52.4%)	8 (22.9%)	0.76
3	5 (23.8%)	5 (14.3%)	0.47
4	9 (43%)	2 (5.7%)	0.18

Supplementary Table 2. Demographics' characteristics of patients that were alive or dead at the time of the analysis, as well as metastatic status before immunotherapy treatment, initial melanoma lesion and initial cancer staging.

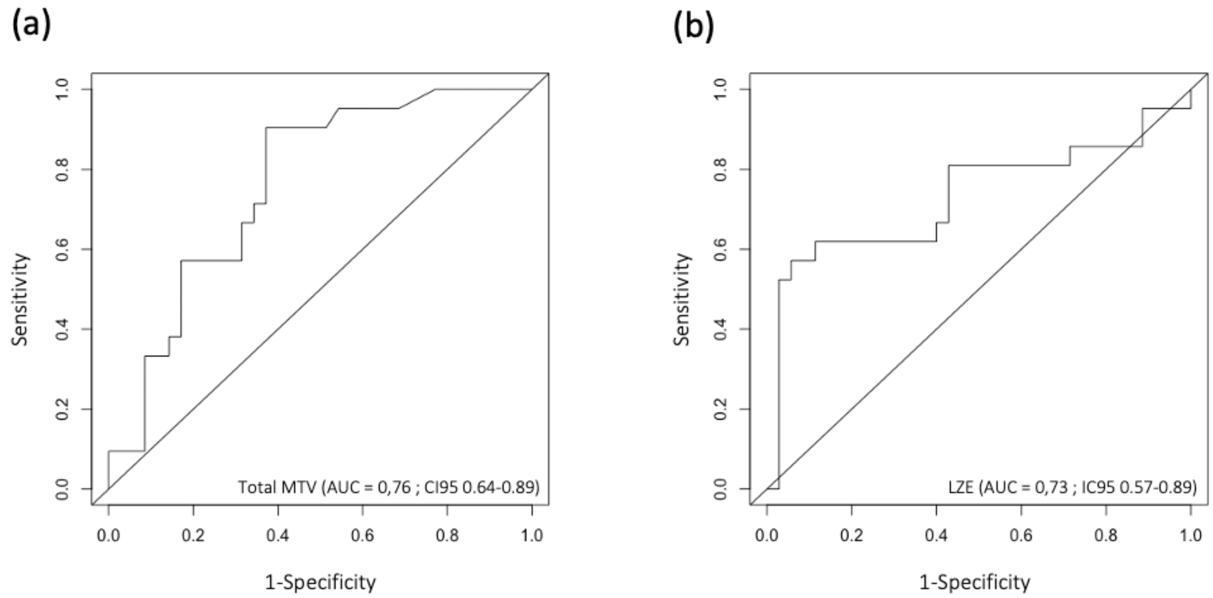

Supplementary Figure 1. Receiver Operating Characteristics curve for overall survival prediction in patients with metastatic melanoma for total metabolic tumoral volume (total MTV) (a) and for long zone emphasis (LZE) (b).

AUC: Area under the curve; CI: confidence interval

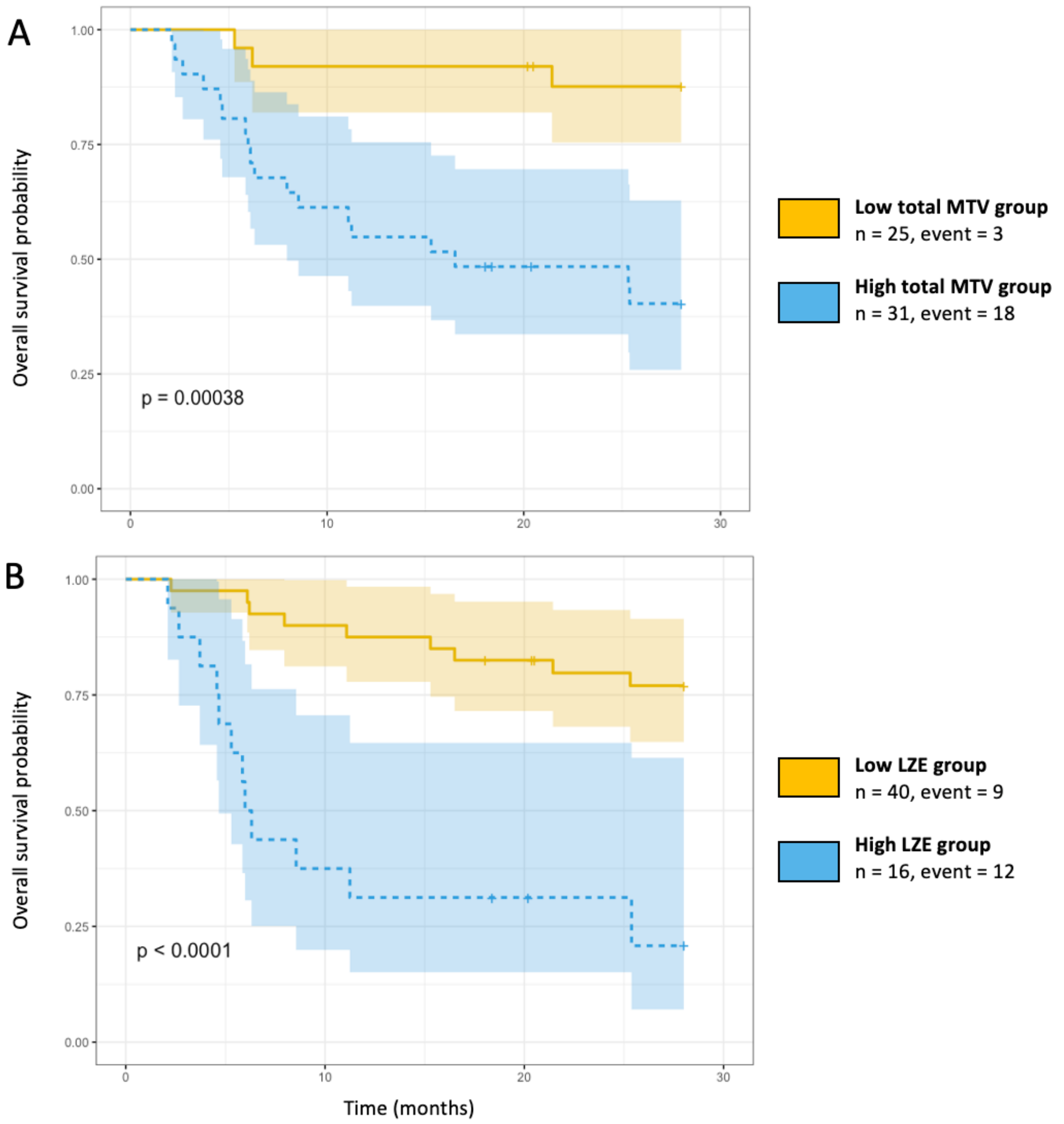

Supplementary Figure 2. Kaplan Meier curves of overall survival according to total metabolic tumour volume (MTV) (A) and long zone emphasis (LZE) values (B). Binary variables cut-offs were total MTV > 5.6cm3 and LZE > -437.

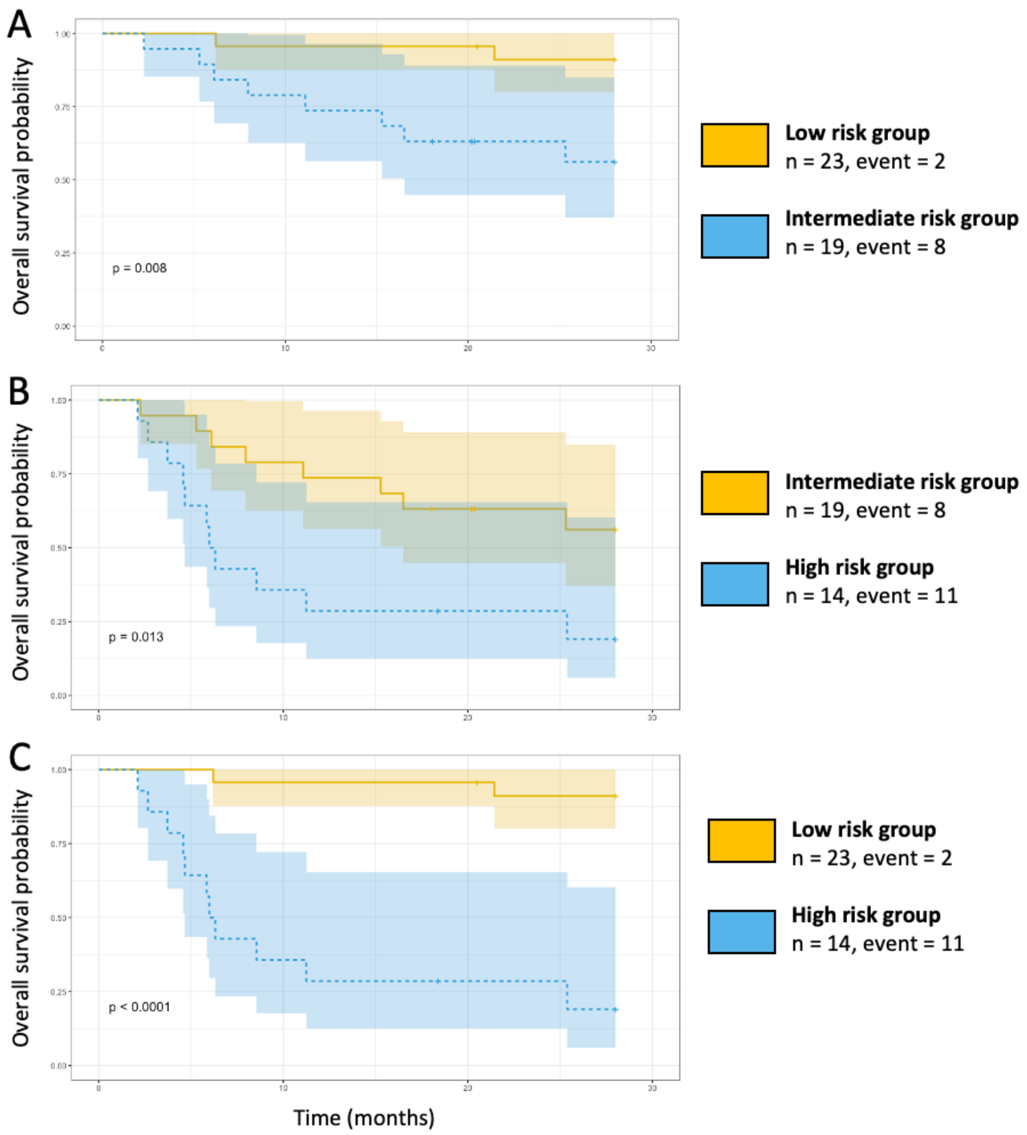

Supplementary Figure 3. Kaplan Meier curves of overall survival according to risk groups based on the metabolic score. A represents the low-risk group versus the intermediate risk group. B represents the intermediate -risk group versus the high-risk group. C represents the low-risk group versus the high-risk group.

The metabolic score comprising two binary risk variables was defined as follows: 0 risk variable = low risk group (total MTV ≤ 5.6cm3 and LZE ≤ -437), 1 risk variable = intermediate risk group (total MTV > 5.6cm3 or LZE > -437) and 2 risk variables = high risk group (total MTV > 5.6cm3 and LZE > -437)

Total MTV: total metabolic tumoral volume, LZE: Long Zone Emphasis
